# Supplementary material for: Yiai Fuzheng decoction inhibits triple-negative breast cancer by remodeling the immune microenvironment
Source: Front Immunol. 2025 Sep 30;16:1615631. doi: 10.3389/fimmu.2025.1615631 (PMC12518410; doi:10.3389/fimmu.2025.1615631)
Supplement: Supplementary file 2 [file DataSheet1.zip › Supplementary File 2/Treatment/C_vs_H/07_Pathway_Analysis/Enrichment_Analysis/smpdb_pathway/Network.pdf]

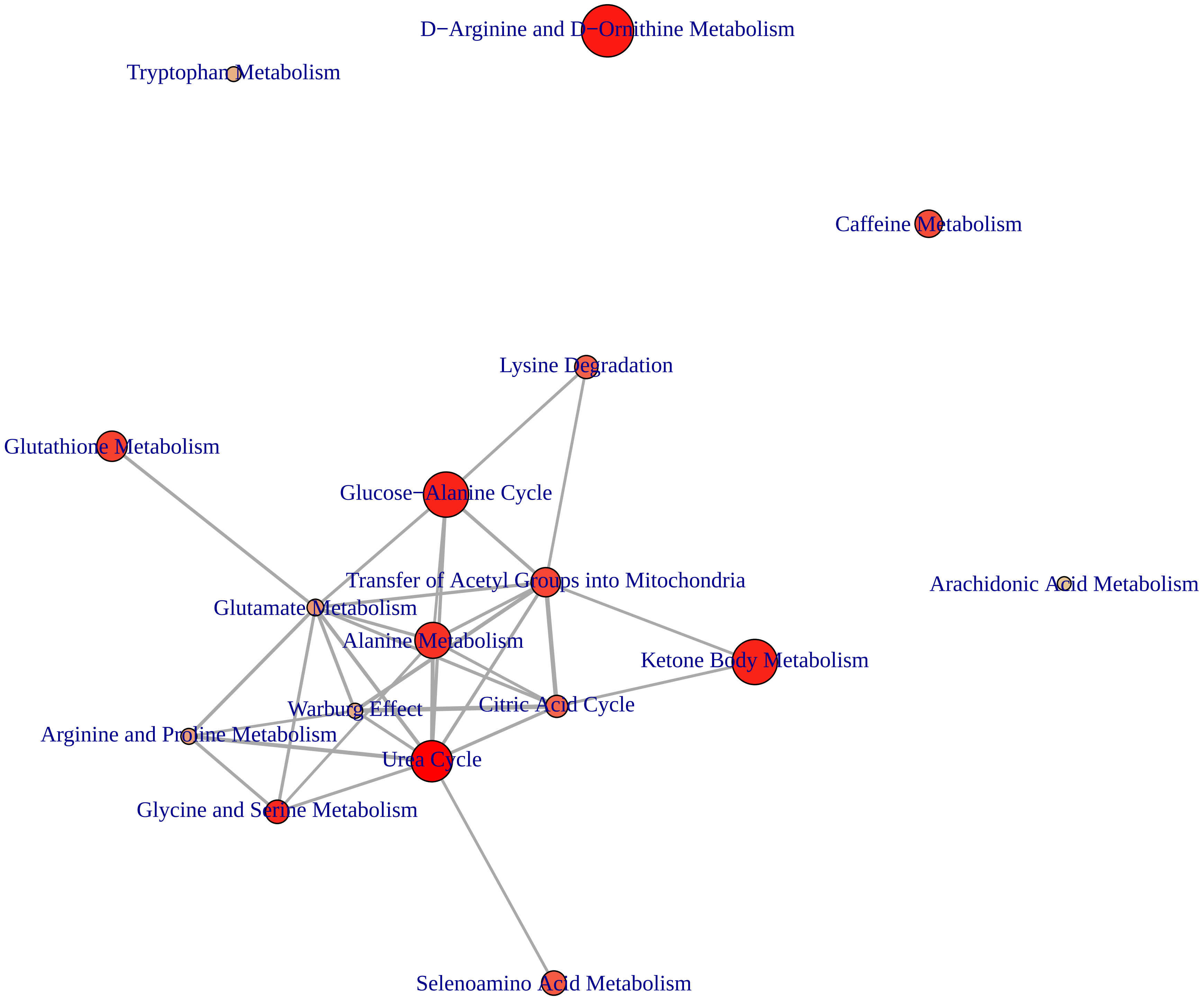
D−Arginine and D−Ornithine Metabolism
Tryptophan Metabolism
Caffeine Metabolism
Lysine Degradation
Glutathione Metabolism
Glucose−Alanine Cycle
Transfer of Acetyl Groups into Mitochondria
Arachidonic Acid Metabolism
Glutamate Metabolism
Alanine Metabolism
Ketone Body Metabolism
Warburg Effect
Citric Acid Cycle
Arginine and Proline Metabolism
Urea Cycle
Glycine and Serine Metabolism
Selenoamino Acid Metabolism
